# Prognostic implication of EGFR gene mutations and histological classification in patients with resected stage I lung adenocarcinoma

**DOI:** 10.1371/journal.pone.0186567

**Published:** 2017-10-24

**Authors:** Chun-Yu Lin, Yen-Mu Wu, Meng-Heng Hsieh, Chih-Wei Wang, Ching-Yang Wu, Ying-Jen Chen, Yueh-Fu Fang

**Affiliations:** 1 Department of General Medicine & Geriatrics, Chang Gung Memorial Hospital at Linkou, Taoyuan, Taiwan; 2 Department of Thoracic Medicine, Chang Gung Memorial Hospital at Linkou, Taoyuan, Taiwan; 3 College of Medicine Chang Gung University, Taoyuan, Taiwan; 4 Department of Infectious Diseases, Department of Internal Medicine, Chang Gung Memorial Hospital Linkou Medical Center, Taoyuan, Taiwan; 5 Graduate Institute of Clinical Medical Sciences, Chang Gung University, Taoyuan, Taiwan; 6 Department of Pathology, Chang Gung Memorial Hospital at Linkou, Taoyuan, Taiwan; 7 Department of Thoracic and Cardiovascular Surgery, Division of Surgery, Chang Gung Memorial Hospital, Linkou, Taiwan; 8 Department of Pulmonary and Critical Care, Saint Paul's Hospital, Taoyuan, Taiwan; National Cancer Center, JAPAN

## Abstract

**Introduction:**

The prognostic value of epidermal growth factor receptor (EGFR) mutations and the correlation between EGFR mutations and the new International Association for the Study of Lung Cancer/American Thoracic Society/European Respiratory Society (IASLC/ATS/ERS) histological classification remain controversial. The current study aimed to investigate the pure prognostic role of EGFR mutations in treatment-naïve patients with resected stage I lung adenocarcinoma.

**Methods:**

We retrospectively reviewed 373 patients with stage I pulmonary non-small-cell lung cancer who underwent complete surgical resection between January 2010 and May 2014. The tumors were classified according to IASLC/ATS/ERS criteria. EGFR mutation status was determined by established methods.

**Results:**

A total of 120 patients were included for analysis; 87 had tumors with EGFR mutations and 33 had wild-type tumors. More low- and intermediate-grade tumors had EGFR mutations, and nearly half of the high-grade tumors were wild-type (75.7% versus 46.2%, p = 0.041). Patients with low-grade tumors had significantly greater median disease-free survival (DFS) (76.8 versus 13 months, p < 0.0001) and better overall survival (OS) (median OS not reached, p = 0.0003) than those with intermediate- and high-grade tumors. Tumor recurrence was 41.4% and 30.3% in mutant and wild-type patients. The 5-years survival rate was 54% and 71.2%. Multivariate analysis revealed that the new histological classification and the pathologic stage were independent predictors of both DFS and OS. EGFR mutation status had no prognostic implications.

**Conclusion:**

Low grade tumors according to IASLC/ATS/ERS histological classification and the pathologic stage IA tumors of resected stage I lung adenocarcinomas independently predict better DFS and OS. EGFR mutations were frequently seen in histologically low- and intermediate-grade tumors but not a prognostic factor.

## Introduction

Lung cancer is the major cause of cancer-related death worldwide [1]. Complete surgical resection remains the best curative treatment for patients with early-stage non-small-cell lung cancer (NSCLC) [2]. Several driver gene mutations are critical for cellular proliferation and survival, including epidermal growth factor receptor (EGFR), echinoderm microtubule-associated protein-like 4 (EML-4), and anaplastic lymphoma kinase (ALK) fusion, which are often highly susceptible to small-molecule tyrosine kinase inhibitors (TKIs) [3–5]. Patients with advanced NSCLC who harbored EGFR mutations consisting of deletion in exon 19 or the L858R mutation in exon 21 had a high response rate and prolonged survival after EGFR-TKI treatment [4, 6–8]. These results indicate that EGFR mutations are the major predictors of response to EGFR-TKI.

Unlike the predictive role of gene mutation in EGFR-TKI therapy, the prognostic value of EGFR mutation status has remained controversial, with conflicting results until now [9–22]. The outcome may not be appropriate in many studies of the prognostic implications of EGFR mutations because of the survival benefit of EGFR-TKI therapy in patients with EGFR mutant tumors. Patients with advanced EGFR mutant NSCLC may also have a better response to chemotherapy than those with wild-type tumors [23]. A new International Association for the Study of Lung Cancer/American Thoracic Society/European Respiratory Society (IASLC/ATS/ERS) histological classification has been validated with prognostic significance [24]. The correlations between genetic alteration and histological subtypes in lung adenocarcinoma remains controversial.

Considering the potential interference of perioperative therapy, the current study focuses on the pure prognostic impact of EGFR mutations in patients with resected stage I lung adenocarcinoma. We also investigate correlations between IASLC/ATS/ERS histological classification and EGFR mutation status [25].

## Materials and methods

### Patients

This retrospective study was approved by our institutional review board (201700457B0). The tissue specimens for genetic analysis were obtained with the patients’ consent. Between January 2010 and May 2014, a total of 373 patients with stage I pulmonary NSCLC underwent complete surgical resection at Linkou Chang Gung Memorial Hospital. Among them, 186 patients were tested for EGFR mutation status at the same time as routine pathologic review of the resected specimen. We excluded patients with stage II, III, or IV NSCLC; those who had positive surgical margins, tumor histological findings other than adenocarcinoma, or absent or failed EGFR genotyping; and those who received neoadjuvant or adjuvant therapy. The final cohort consisted of 120 patients.

### Histology and EGFR mutation analysis

The resected specimens were formalin-fixed and stained with hematoxylin and eosin in the conventional manner. Each tumor was reviewed using comprehensive histological subtyping by semiquantitatively estimating the percentages of the various subtypes present in 5% increments according to the IASLC/ATS/ERS classification [25]. The predominant pattern was defined as the pattern showing the greatest percentage, which was not necessarily 50% or greater. Each pulmonary adenocarcinoma subtype was further categorized as low-grade (adenocarcinoma in situ, minimal invasive adenocarcinoma, or lepidic predominant adenocarcinoma), intermediate-grade (acinar/papillary predominant adenocarcinoma), or high-grade (solid/micropapillary predominant, invasive mucinous adenocarcinoma) based on the prognosis [24, 26]. EGFR mutations were detected by direct DNA sequencing when the tumor purity was greater than 40%, and Scorpion amplification-refractory mutation system when the tumor purity was 5 to 40%. Competitive allele-specific TaqMan polymerase chain reaction was chosen when the tumor purity was below 5% [27]. EGFR mutations other than exon 19 deletion or exon 21 L858R mutation were categorized as rare mutations.

### Covariates and data collection

Data on baseline patient characteristics were collected, including age, sex, smoking history, and Eastern Cooperative Oncology Group (ECOG) performance status. The date and type of surgery were recorded. Tumor stage was categorized according to the seventh edition of the American Joint Committee on Cancer guidelines for NSCLC [28]. After the operation, the patients were followed with chest X-rays every 3 months in the first year, every 6 months for the next 2 years, and yearly thereafter. Chest to abdomen computed tomography (CT) scans were obtained at least annually. Tumor recurrence was defined as radiographic evidence of cancer relapse on surveillance imaging, such as enlarging opacity at primary site, sequential enlarging opacity, bulging margin or loss of linear margin, etc. If possible, recurrences were confirmed pathologically. Disease-free survival (DFS) was defined as the time from initial surgery until tumor recurrence. Overall survival (OS) was defined as the time from initial surgery until death. The dates and causes of death were obtained from medical records.

### Statistical analysis

The patients were divided into two groups, EGFR mutant and wild-type, based on the presence or absence of EGFR. Associations between clinical characteristics and recurrence rates were examined by Fisher’s exact test. DFS and OS were analyzed by the Kaplan—Meier method, and survival curves were compared by the log rank test. Independent prognostic factors for DFS and OS were determined by Cox multivariate analysis. Statistical significance was set at p < 0.05. The statistical analyses were performed with GraphPad Prism statistical software, version 6 (GraphPad Software, La Jolla, CA, USA) and IBM SPSS Statistics 20 for Mac (SPSS, Chicago, IL, USA).

## Results

### Patients

The characteristics of the 120 patients and their tumors are summarized in Table 1. There were more women (63.2% versus 30.3%, p = 0.002) and never-smokers (81.6% versus 60.6%, p = 0.009) in the EGFR mutant group than in the wild-type group. Most patients (102 of 120 [85%]) underwent lobectomy, and the surgical procedures did not differ between the EGFR mutant group and the wild-type group. Tumor size and pathologic stage were comparable between the EGFR mutant group and the wild-type group. Half of the patients (67 of 120 [55.8%]) had lepidic predominant adenocarcinoma. More low- and intermediate-grade tumors than high-grade tumors had EGFR mutations (81 of 107 [75.7%] versus 6 of 13 [46.2%]; p = 0.041). More patients with lepidic predominant subtype (51 of 67 [76.1%]) or acinar subtype (26 of 31 [83.9%]) tumors had EGFR mutations than patients with wild-type tumors; however, the differences were not significant (p = 0.41 for lepidic subtype, p = 0.11 for acinar subtype).

**Table 1:** Demographics of patients and tumor characteristics.

	EGFR mutant	Wild-type	
Variable	*n* = 87	*n* = 33	*p* Value
Age (y), mean ± SD	62.9 ± 1.3	64.2 ± 2.2	0.60
Sex, *n* (%)			0.002
Male	32 (36.8)	23 (69.7)	
Female	55 (63.2)	10 (30.3)	
Smoking, *n* (%)			0.009
Never	71 (81.6)	20 (60.6)	
Ever	16 (18.4)	13 (39.4)	
ECOG score, *n* (%)			1.0
0	79 (90.8)	30 (90.9)	
1	8 (9.2)	3 (9.1)	
Tumor size (cm), *n* (%)			0.30
≤ 2	28 (32.2)	13 (39.4)	
2–3	47 (54.0)	14 (42.4)	
> 3	12 (13.8)	6 (18.2)	
Pleural involvement, *n* (%)			0.64
Invasion	23 (26.4)	7 (21.2)	
Free	64 (73.6)	26 (78.8)	
Stage, *n* (%)			0.83
IA	57 (65.5)	23 (69.7)	
IB	30 (34.5)	10 (30.3)	
Histological classification, *n* (%)			0.21
Low-grade	51 (58.6)	18 (54.5)	
Adenocarcinoma in situ	0 (0)	2 (6.1)	
Lepidic	51 (58.6)	16 (48.5)	
Intermediate-grade	30 (34.5)	8 (24.2)	
Acinar	26 (29.9)	5 (15.2)	
Papillary	4 (4.6)	3 (9.1)	
High-grade	6 (6.9)	7 (21.2)	
Micropapillary	1 (1.1)	2 (6.1)	
Solid	5 (5.7)	4 (12.1)	
Invasive mucinous	0 (0)	1 (3.0)	
EGFR mutation, *n* (%)			
Exon 19 deletion	31 (35.6)		
Exon 21 L858R	52 (59.8)		
Rare mutation	4 (4.6)		
Surgical procedure, *n* (%)			0.19
Lobectomy	75 (86.2)	27 (81.8)	
Segmentectomy	0 (0)	1 (3)	
Wedge resection	12 (13.8)	5 (15.2)	
Recurrence, *n* (%)	36 (41.4)	10 (30.3)	0.30
Free, *n* (%)	51 (58.6)	23 (69.7)	

ECOG, Eastern Cooperative Oncology Group; EGFR, epidermal growth factor receptor.

### Genotype results

Among the EGFR mutant group, 84 patients had a single mutation and 3 patients had double mutations. The most frequent EGFR mutations were the exon 21 point mutation L858R in 52 patients (59.8%) and the exon 19 deletion in 31 patients (35.6%). All of the three patients with double mutations had L858R. One of these patients had the exon 20 mutation T790M, one had R776H, and one had the exon 21 mutation K806I. The remaining four patients had rare mutations, including one patient with exon 20 insertion mutation, one with exon 20 deletion, one with exon 18 G724S, and one with exon 18 G719X.

### Analyses of disease-free survival and overall survival

**Fig 1:**
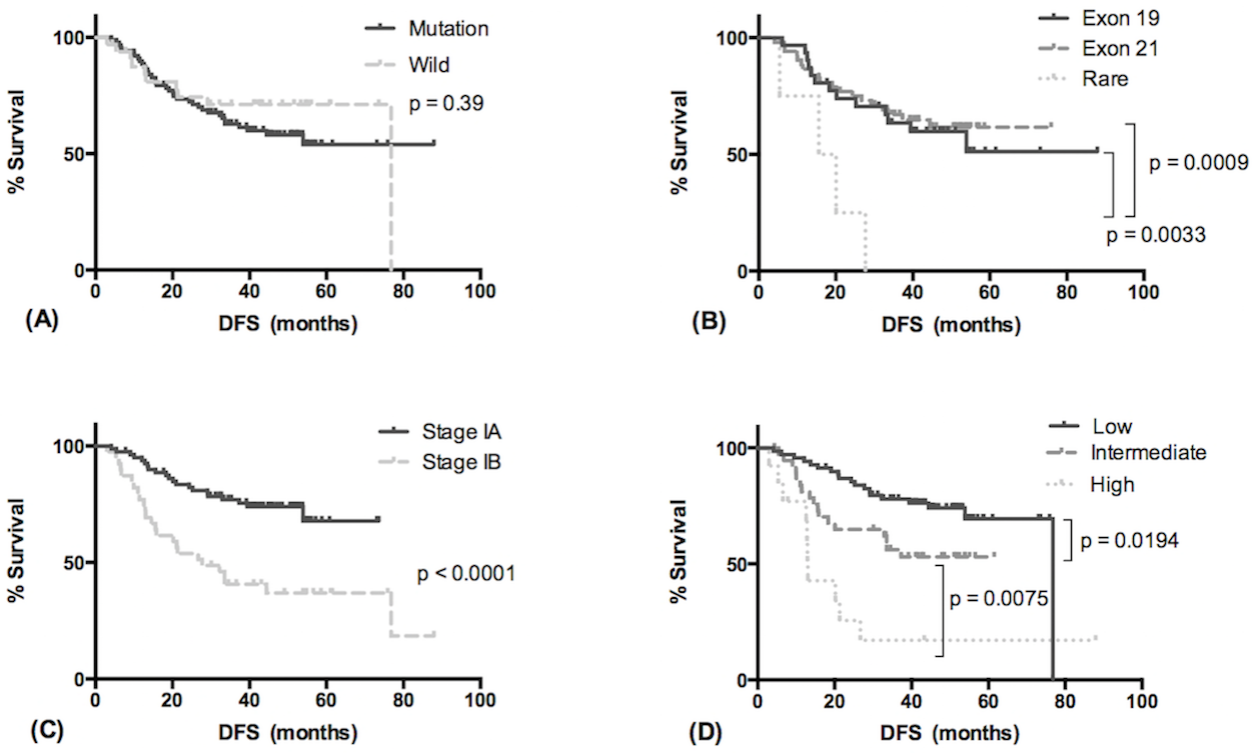
Disease-free survival of patients with stage I resected pulmonary adenocarcinoma. (A, B) Disease-free survival in relation to EGFR mutation status. (C) Disease-free survival in relation to tumor pathologic stage. (D) Disease-free survival in relation to tumor histological classification.

The median follow-up time was 46.7 months. Tumor recurrence was observed in 46 patients (36 [41.4%] in the EGFR mutant group and 10 [30.3%] in the wild-type group; p = 0.3). Four patients died of non-lung-cancer-related disease without tumor recurrence (two in the EGFR mutant group and two in the wild-type group). The median DFS was 76.8 months in the EGFR mutant group and was not reached in the wild-type group (p = 0.39) (Fig 1A, Table 2). Fifty-four percent of patients in the EGFR mutant group remained disease-free at 5 years, compared with 71.2% in the wild-type group. In the EGFR mutant subgroup analysis, patients harboring rare mutations had significantly shorter median DFS than those with exon 19 deletions or exon 21 L858R point mutations (17.85 months versus unreached in both the exon 19 and the exon 21 subgroups; p = 0.0033 and 0.0009, respectively) (Fig 1B). Univariate analysis revealed that pathologic stage IA and low-grade histological subtype were favorable prognostic factors for DFS (Table 2). The median DFS was 27.7 months in patients with stage IB tumors and was not reached in patients with stage IA tumors (p < 0.0001) (Fig 1C). DFS was significantly correlated with tumor histological classification (median DFS: 13 months in the high-grade, unreached in the intermediate-grade, and 76.8 months in the low-grade group; p < 0.0001) (Fig 1D). Sex, smoking status, types of surgical procedure and EGFR mutation status were not significant. Only pathologic stage (stage IA versus stage IB: hazard ratio [HR], 0.414; 95% confidence interval [CI], 0.223–0.955; p = 0.005) and tumor histological classification (low- versus intermediate- and high-grade: HR, 0.492; 95% CI, 0.262–0.917; p = 0.026) were independently significant predictors for DFS in the Cox proportional hazards model.

**Table 2:** Cox proportional hazards model for analysis of disease-free survival.

		Univariate analysis			Multivariate analysis		
Variable	Category	HR	95%CI	*P* value	HR	95%CI	*P* value
Sex	Male/female	1.610	0.9061–2.917	0.1039			
Smoking	Ever/never	0.9400	0.4711–1.877	0.8622			
Surgical procedure	Lobectomy/Wedge resection	0.6326	0.2484–1.360	0.2123	0.489	0.230–1.041	0.064
Pathologic stage	IA/IB	0.3445	0.1433–0.511	< 0.0001	0.382	0.206–0.709	0.002
Histology	Low/intermediate, high	0.3923	0.196–0.668	0.0012	0.465	0.252–0.859	0.015
EGFR mutation	Mutant/wild	1.353	0.6939–2.539	0.3941			

CI, confidence interval; EGFR, epidermal growth factor receptor; HR, hazard ratio.

Treatment after disease recurrence is summarized in Table 3. Of the 36 patients in the EGFR mutant group with recurrence, 19 (52.8%) were treated with EGFR TKI, 15 (41.7%) received systemic chemotherapy, 7 (19.4%) underwent surgery, 3 (8.3%) received radiotherapy, and 2 (5.6%) received supportive care because of poor performance status. In the wild-type group, four patients (40%) were treated with EGFR TKI, five (50%) received systemic chemotherapy, two (20%) underwent surgery, one (10%) received radiotherapy, and one (10%) received immunotherapy.

**Table 3:** Treatment modalities for recurrent disease.

Therapy after recurrence	EGFR mutant *n* = 36	Wild-type *n* = 10
EGFR TKI, *n* (%)	19 (52.8)	4 (40)
Chemotherapy, *n* (%)	15 (41.7)	5 (50)
Surgery, *n* (%)	7 (19.4)	2 (20)
Radiotherapy, *n* (%)	3 (8.3)	1 (10)
Immunotherapy, *n* (%)	0 (0)	1 (10)
Supportive care, *n* (%)	2 (5.6)	0 (0)

EGFR, epidermal growth factor receptor; TKI, tyrosine kinase inhibitor.

**Fig 2:**
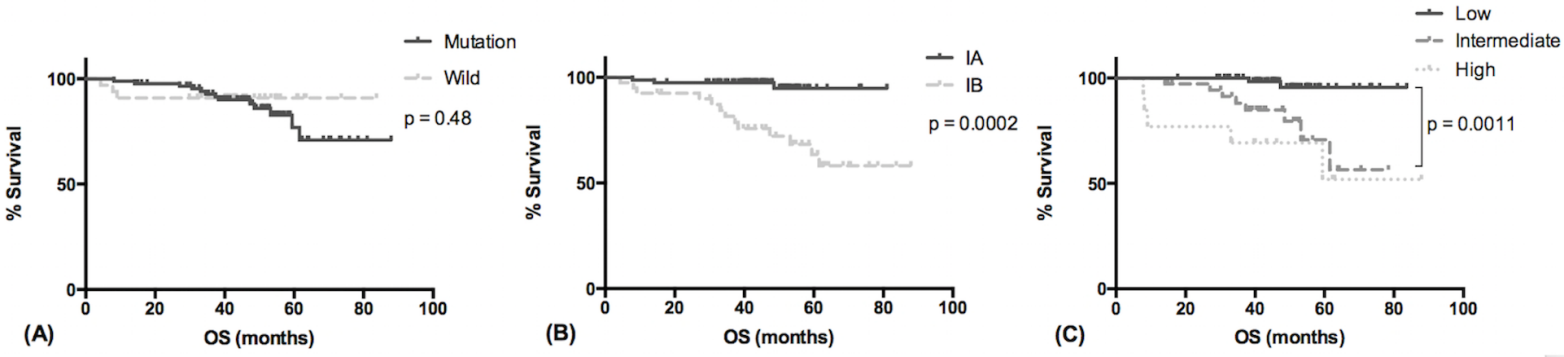
Overall survival of patients with stage I resected pulmonary adenocarcinoma. (A) Overall survival in relation to EGFR mutation status. (B) Overall survival in relation to tumor pathologic stage. (C) Overall survival in relation to histological classification.

There were 16 deaths, 13 in the EGFR mutant group and 3 in the wild-type group (p = 0.55). The median OS was unreached in both groups, and OS was comparable between groups (HR, 1.567; 95% CI, 0.4923–4.528; p = 0.4792). The estimated 5-year survival rate was 78% in the EGFR mutant group and 96.8% in the wild-type group (Fig 2A) (p = 0.48). Although the median OS was not reached, patients with stage IA tumor and those with low-grade adenocarcinomas had better OS (p = 0.0002, Fig 2B; p = 0.0003, Fig 2C). The prognostic value of sex, smoking status, types of surgical procedure and EGFR mutation status were not significant. In the Cox proportional hazards model, pathologic stage (stage IA versus stage IB: HR, 0.214; 95% CI, 0.058–0.788; p = 0.02) and histological classification (low- versus intermediate- and high-grade: HR, 0.151; 95% CI, 0.033–0.687; p = 0.015) were also independent prognostic factors for OS (Table 4).

**Table 4:** Cox proportional hazards model for analysis of overall survival.

		Univariate analysis			Multivariate analysis		
Variable	Category	HR	95%CI	*P* value	HR	95%CI	*P* value
Sex	Male/female	1.232	0.4608–3.307	0.6758			
Smoking	Ever/never	1.047	0.3334–3.291	0.9367			
Surgical procedure	Lobectomy/Wedge resection	3.019	0.5732–7.906	0.2598	1.934	0.248–15.088	0.529
Pathologic stage	IA/IB	0.1413	0.050–0.3773	0.0002	0.222	0.060–0.824	0.024
Histology	Low/intermediate, high	0.1008	0.0491–0.3894	0.0003	0.164	0.035–0.768	0.022
EGFR mutation	Mutant/wild	1.567	0.4923–4.528	0.4792			

CI, confidence interval; EGFR, epidermal growth factor receptor; HR, hazard ratio.

## Discussion

To our knowledge, this is the first study to focus on the prognostic difference by EGFR genotype and IASLC/ATS/ERS histological classification in resected stage I pulmonary adenocarcinoma. We investigated the outcomes in a cohort of 120 patients who had known EGFR mutation status and no perioperative therapy. We found that EGFR mutation status was unrelated to DFS and OS. EGFR mutations were more frequent in patients with low- and intermediate-grade tumors. Pathologic stage and IASLC/ATS/ERS histological classification were independent prognostic factors for both DFS and OS in patients with resected stage I pulmonary adenocarcinoma.

There is accumulating evidence for a prognostic role of EGFR gene mutations in patients with resected NSCLC [9, 13, 14, 16, 20, 22, 29, 30]. However, many studies included patients with resected lung tumors in different stages and a variety of treatments. The lower risk of death in patients with EGFR mutations may be due to the survival benefit of EGFR-TKI therapy [16, 21]. Moreover, EGFR mutant tumors are more sensitive to chemotherapy than are wild-type tumors [23]. It is difficult to determine the outcome from disease biology or the benefit from perioperative therapy. The heterogeneity of treatment for recurrent disease may also affect OS [21]. DFS reflects the intrinsic biologic malignancy of EGFR mutation status. Thus, using DFS rather than OS and focusing on stage I patients who underwent resection without perioperative treatment is more appropriate for investigating the prognostic role of EGFR mutations. Five articles have focused on the prognostic implications of EGFR mutations in patients with resected stage I NSCLC [13, 14, 18, 20, 30].

Izar et al. found that patients with resected stage I, EGFR mutation-positive NSCLC without adjuvant therapy had significantly better DFS and OS than those with wild-type tumors [20]. Only 62 patients in the study had EGFR mutant tumors, and the other 245 patients had wild-type tumors because of the racial difference. In addition to EGFR mutations, KRAS gene mutations, which are responsible for NSCLC in 30% of white patients and 10%–15% of Asian patients, are also a prognostic factor [12, 31]. Among patients with resected stage I–III tumors, those with KRAS mutations had worse OS than those with EGFR mutations [15]. Another retrospective study of 312 patients with resected stage I lung adenocarcinoma conducted by Izar et al. showed that patients who had KRAS mutations had worse OS and DFS than patients who had EGFR mutations or were wild-type for both KRAS and EGFR [32]. In two Japanese studies, Ohba et al. found that patients with EGFR mutations and those with wild-type tumors had similar DFS and OS. KRAS mutations were the only independent prognostic factor in patients with resected stage I adenocarcinoma [13, 14]. In the Japanese studies, 41%–47% of patients had EGFR mutations and 5% had KRAS mutations. In contrast, in the study conducted by Izar et al [32]., only 19% of patients had EGFR mutations and 40% had KRAS mutations. The conflicting results concerning the implication of EGFR mutations for prognosis may be due to the racial difference. Because of the very low prevalence of KRAS mutations in Taiwan, we did not test for KRAS mutations regularly [10, 30]. Nevertheless, similar to the findings of Ohba et al., we found no difference in DFS and OS between patients with EGFR mutant tumors and those with wild-type tumors.

Kobayashi et al. showed that tumor differentiation was the only independent factor for unfavorable OS and DFS [33]. They also found that the non-bronchioalveolar carcinoma component, but not EGFR mutation status, was strongly associated with poorer outcome [18]. The comprehensive lung adenocarcinoma classification proposed by IASLC/ATS/ERS in 2011 has significant implications for the prognostic role of tumor histology [24, 34]. Many studies have discussed the correlation between tumor gene mutations and histological classification. The strongest correlation between a histological subtype and a set of molecular and biologic features is that of invasive mucinous adenocarcinomas, which typically have KRAS mutations and lack EGFR mutations [25]. In one retrospective study of 410 patients with stage I lung adenocarcinoma, Isaka et al. showed that the EGFR exon 21 mutation was associated with low-grade adenocarcinoma with lepidic predominance, and that wild-type tumors were frequently high-grade tumors with vascular invasion [35]. In our study, we found that more low- and intermediate-grade tumors than high-grade tumors had EGFR mutations (81 of 107 [75.7%] versus 6 of 13 [46.2%]; p = 0.041). Moreover, the prognosis was strongly associated with tumor grade but not EGFR mutation status for both DFS and OS.

Accumulating evidence has validated the prognostic significance of the predominant histological subtype based on the IASLC/ATS/ERS classification rather than gene mutation status [34, 36–38]. Yoshizawa et al. reviewed 440 patients with resected lung adenocarcinomas and found that stage and histological grade were independent prognostic factors for both DFS and OS [38]. EGFR mutations were associated with low- to intermediate-grade adenocarcinomas, and KRAS mutations were associated with the mucinous subtype. However, in that study, KRAS mutations were not a prognostic factor for DFS and OS. Only EGFR mutations predicted a favorable 5-year survival rate, which could have been a benefit of EGFR-TKI treatment or adjuvant chemotherapy. We included only patients with completely resected stage I lung adenocarcinoma who had never received perioperative treatment. Thus, we presented the biologically prognostic values of EGFR mutations and histological classification in patients with adenocarcinoma.

Our study has several inherent limitations. First, it was retrospective in design and conducted in a single center. There were 187 excluded patients who were not genotyped. Sixty-six patients were excluded because they had received perioperative treatment. Thus, the proportion of EGFR mutant tumors is somewhat higher in our study compared with general population in lung cancer patients. Second, the rule of EGFR mutation detection methods in our hospital is based on the tumor purity. Because the sensitivity of detection methods is different, this may somewhat interfere the outcome. Third, we only analyzed the outcome of patients with tumors harboring EGFR mutations and the relationship between EGFR mutations and histology. Because of the rarity of KRAS mutations in Taiwan, we did not investigate the potential influence of these mutations.

## Conclusions

In summary, our study demonstrated that patients with stage IA tumor and those with low-grade tumors had better DFS and OS in patients with resected stage I lung adenocarcinoma who had no perioperative therapy. Although EGFR mutations were more frequently seen in patients with low- and intermediate-grade tumors, they had no prognostic implication. To investigate the prognostic role of driver mutations in lung adenocarcinomas and analyze the molecular correlations with histological subtypes, further prospective studies are warranted of larger numbers of patients, focusing on early-stage, resected lung cancer without perioperative treatment.

## Supporting information

S1 Dataset (XLSX)

## Abbreviations

DFS: disease-free survival
ECOG: Eastern Cooperative Oncology Group
EGFR: epidermal growth factor receptor
IASLC/ATS/ERS: International Association for the Study of Lung Cancer/American Thoracic Society/European Respiratory Society
NSCLC: non-small-cell lung cancer
OS: overall survival
TKI: tyrosine kinase inhibitor
